# Supplementary material for: Multicentennial record of Labrador Sea primary productivity and sea-ice variability archived in coralline algal barium
Source: Nat Commun. 2017 Jun 1;8:15543. doi: 10.1038/ncomms15543 (PMC5461504; doi:10.1038/ncomms15543)
Supplement: Supplementary Information — Supplementary Figure, Supplementary Note and Supplementary References [file ncomms15543-s1.pdf]

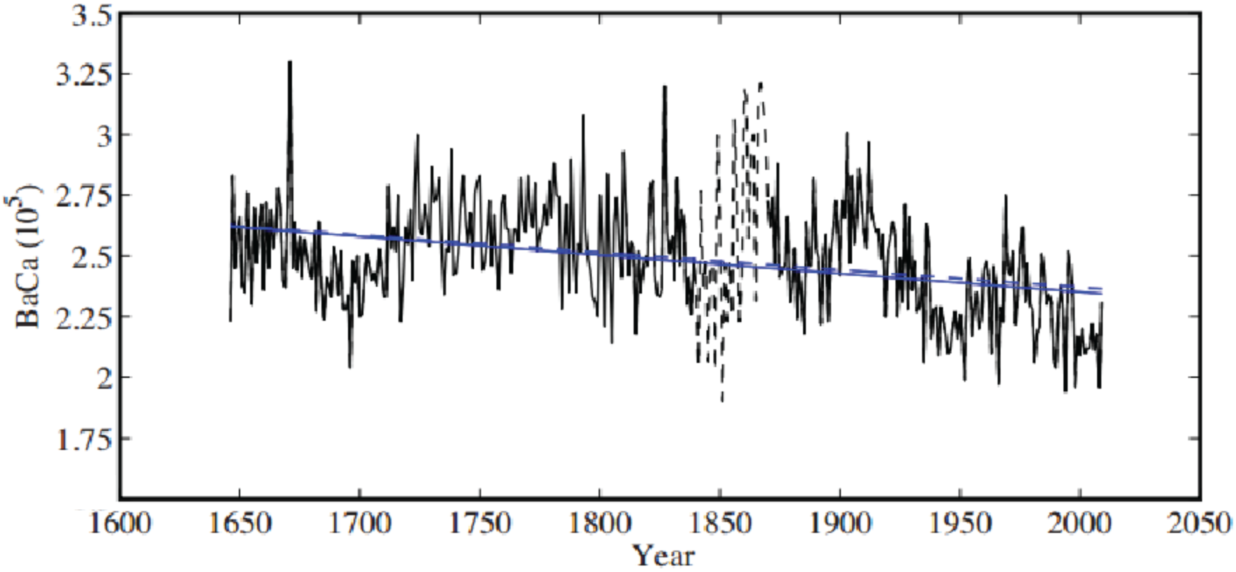

Supplementary Figure 1 | Singular Spectral Analysis (SSA) of time series. Annually averaged Ba/Ca time series (solid) and the gap-filled Ba/Ca time series (dashed) from 1646 to 2009. The linear trend in gap-filled time series did not significantly differ from the unfilled time series (significant at the 99% level). Linear least squares regression fits both time series (blue). The detrended gap-filled Ba/Ca record was used for correlations to the proxy AMO index.

Supplementary Note 1

Crustose Coralline Algae

Crustose coralline algae are calcareous photoautotrophic marine algae that are abundant and geographically widespread, forming hard rock-like encrustations in shallow rocky sublittoral zones worldwide[3]. Coralline algae of the genus *Clathromorphum* form annual growth increments, and are exceptionally abundant in subarctic regions of the North Atlantic, North Pacific, and Arctic Oceans. In the Northwest Atlantic, the coralline algae can form accretions of up to 10.5 cm in thickness (at an average vertical extension rate of 170 µm/year), resulting in age spans of several hundred years[4]. In fact, a living specimen of *Clathromorphum compactum* collected from the northern Labrador, Canada has been dated using annual layer counting methods, backed by radiocarbon dating to an age of 646 years[4]. Thus, as a result of their abundance, distribution, longevity, and formation of clear and distinct annual growth increments; coralline algae have become excellent high-resolution paleoclimate archives for reconstructing past changes in the ocean environment.

Over the past decade, a number of different geochemical methods have been utilized to examine: stable oxygen isotopes ($\delta^{18}O$)[5,6], trace element compositions (ex. Mg/Ca, Ba/Ca, Sr/Ca, U/Ca)[7,8,9] and growth increment widths[10]. Proxy data obtained from *Clathromorphum* have been calibrated to in-situ water temperatures in a year-long field study[6], and inter and intra-specimen comparisons of geochemical data have demonstrated high reproducibility[7, 11]. This wealth of proxy information spanning multidecadal to multicentennial timescales has provided us with a better understanding of variations in sea surface temperature[9, 12, 13], cloud cover and shallow marine light dynamics[10, 14], Arctic sea-ice cover[4]; and their associations with large-scale multi-

decadal climate oscillation patterns (ex. Pacific Decadal Oscillation, North Atlantic Oscillation, El Niño Southern Oscillation, Aleutian Low) that were previously unresolvable due to the short record of oceanographic observations.

Supplementary References

1. Swart, P. K., *et al.* The ^{13}C Suess effect in scleractinian corals mirror changes in the anthropogenic CO_2 inventory of the surface oceans. *Geophys. Res. Lett.* **37,** L05604 (2010).

2. Good, S. A., Martin, M. J. & Rayner, N. A. EN4: Quality controlled ocean temperature and salinity profiles and monthly objective analyses with uncertainty estimates. *Journal of Geophysical Research: Oceans* **118,** 6704-6716 (2013).

3. Steneck, R. S. The ecology of coralline algal crusts: Convergent patterns and adaptative strategies. *Annu. Rev. Ecol. Syst.* **17,** 273-303 (1986).

4. Halfar, J., *et al.* Arctic sea-ice decline archived by multicentury annual-resolution record from crustose coralline algal proxy. *Proc. Natl. Acad. Sci. U. S. A.* **110,** 19737-19741 (2013).

5. Halfar, J., *et al.* Coralline alga reveals first marine record of subarctic North Pacific climate change. *Geophys. Res. Lett.* **34,** L07702 (2007).

6. Halfar, J., *et al.* Coralline red algae as high-resolution climate recorders. *Geology* **36,** 463-466 (2008).

7. Hetzinger, S., *et al.* High-resolution analysis of trace elements in crustose coralline algae from the North Atlantic and North Pacific by laser ablation ICP-MS. *Paleogeogr. Paleoclimatol. Paleoecol.* **302,** 81-94 (2011).

8. Chan, P., *et al.* Freshening of the Alaska Coastal Current recorded by coralline algal Ba/Ca ratios. *J. Geophys. Res. Biogeo.* **116,** G01032 (2011).

9. Gamboa, G., *et al.* Mg/Ca ratios in coralline algae record northwest Atlantic temperature variations and North Atlantic Oscillation relationships. *J. Geophys. Res. Oceans* **115,** C12044 (2010).

10. Halfar, J., *et al.* 225 years of Bering Sea climate and ecosystem dynamics revealed by coralline algal growth-increment widths. *Geology* **39,** 579-582 (2011).

11. Hetzinger, S., *et al.* Coralline algal barium as indicator for 20th century northwestern North Atlantic surface ocean freshwater variability. *Sci. Rep.* **3,** 1761 (2013).

12. Kamenos, N. A., Cusack, M. & Moore, P. G. Coralline algae are global palaeothermometers with bi-weekly resolution. *Geochim. Cosmochim. Acta* **72,** 771-779 (2008).

13. Hetzinger, S., *et al.* High-resolution Mg/Ca ratios in a coralline red alga as a proxy for Bering Sea temperature variations from 1902 to 1967. *Palaios* **24,** 406-412 (2009).

14. Burdett, H., Kamenos, N. A. & Law, A. Using coralline algae to understand historic marine cloud cover. *Paleogeogr. Paleoclimatol. Paleoecol.* **302,** 65-70 (2011).
